# Adenomatous Hyperplasia of Duodenal Brunner's Glands: An Extremely Uncommon Imitation of Malignant Pathology

**DOI:** 10.7759/cureus.88551

**Published:** 2025-07-22

**Authors:** Archit Garg, Vikram Jeet Singh Gill, Mehak Bassi, Andrew Korman, Arkady Broder

**Affiliations:** 1 Internal Medicine, Saint Peter's University Hospital/Rutgers Robert Wood Johnson Medical School, New Brunswick, USA; 2 Internal Medicine, Saint Peter’s University Hospital/Rutgers Robert Wood Johnson Medical School, New Brunswick, USA; 3 Gastroenterology and Hepatology, Saint Peter's University Hospital/Rutgers Robert Wood Johnson Medical School, New Brunswick, USA; 4 Gastroenterology and Hepatology, Saint Peter’s University Hospital/Rutgers Robert Wood Johnson Medical School, New Brunswick, USA; 5 Gastroenterology and Hepatology, Saint Peter’s University Hospital, New Brunswick, USA

**Keywords:** adenomyoma, brunner's gland hyperplasia, duodenal nodule, esophagogastroduodenoscopy, heartburn

## Abstract

Adenomatous hyperplasia of duodenal Brunner’s glands is a rare benign pathology of the duodenum linked to epigastric pain and dyspepsia. However, in rare cases, it can cause intestinal obstruction. Hence, endoscopic or surgical removal of Brunner’s gland hyperplasia (BGH) has been suggested to prevent complications including hemorrhage, severe anemia due to persistent bleeding, intussusception, and obstruction. BGH can be managed with endoscopic polypectomy. It represents a less invasive alternative to surgery and is more cost-effective. The medical treatment mainly involves treating gastric hyperacidity, a known cause of BGH, but the regression of BGH is rare. This case report aims to describe and investigate the clinicopathologic features of this rare pathology. The case emphasizes the importance of endoscopy for the evaluation of the refractory gastroesophageal reflux-like presentation and demonstrates that histopathological evaluation remains critical for a definitive diagnosis of BGH and to rule out malignancy. Conservative approaches may suffice in select patients, avoiding invasive interventions. Follow-up remains essential to monitor for recurrence or complications.

## Introduction

Adenomatous hyperplasia of the duodenal Brunner's glands is an infrequently encountered benign proliferative condition. It comprises 5-10% of small bowel tumors and typically manifests in individuals between their fifth and sixth decades [1,2]. This condition does not exhibit a significant propensity for any gender or racial group. Most cases are asymptomatic and discovered incidentally. On rare occasions, these growths can induce obstructive symptoms, resulting in postprandial pain and upper gastrointestinal bleeding, necessitating endoscopic treatment or surgical removal [3]. Adenomatous hyperplasia is typically diagnosed through endoscopy and may be discovered incidentally or during evaluation for persistent abdominal pain or upper gastrointestinal bleeding [2-4]. Management depends on the presenting symptoms. Small, asymptomatic lesions can be monitored with periodic surveillance endoscopies to ensure stability in size. In symptomatic patients, gastric acid suppression with proton pump inhibitors (PPIs) may provide relief [3,4]. For enlarging lesions or those causing complications such as bleeding or obstruction, endoscopic removal is recommended, with biopsies performed to exclude malignancy [4,5]. Here, we present a unique case of adenomatous hyperplasia involving Brunner's glands.

## Case presentation

**Figure 1:**
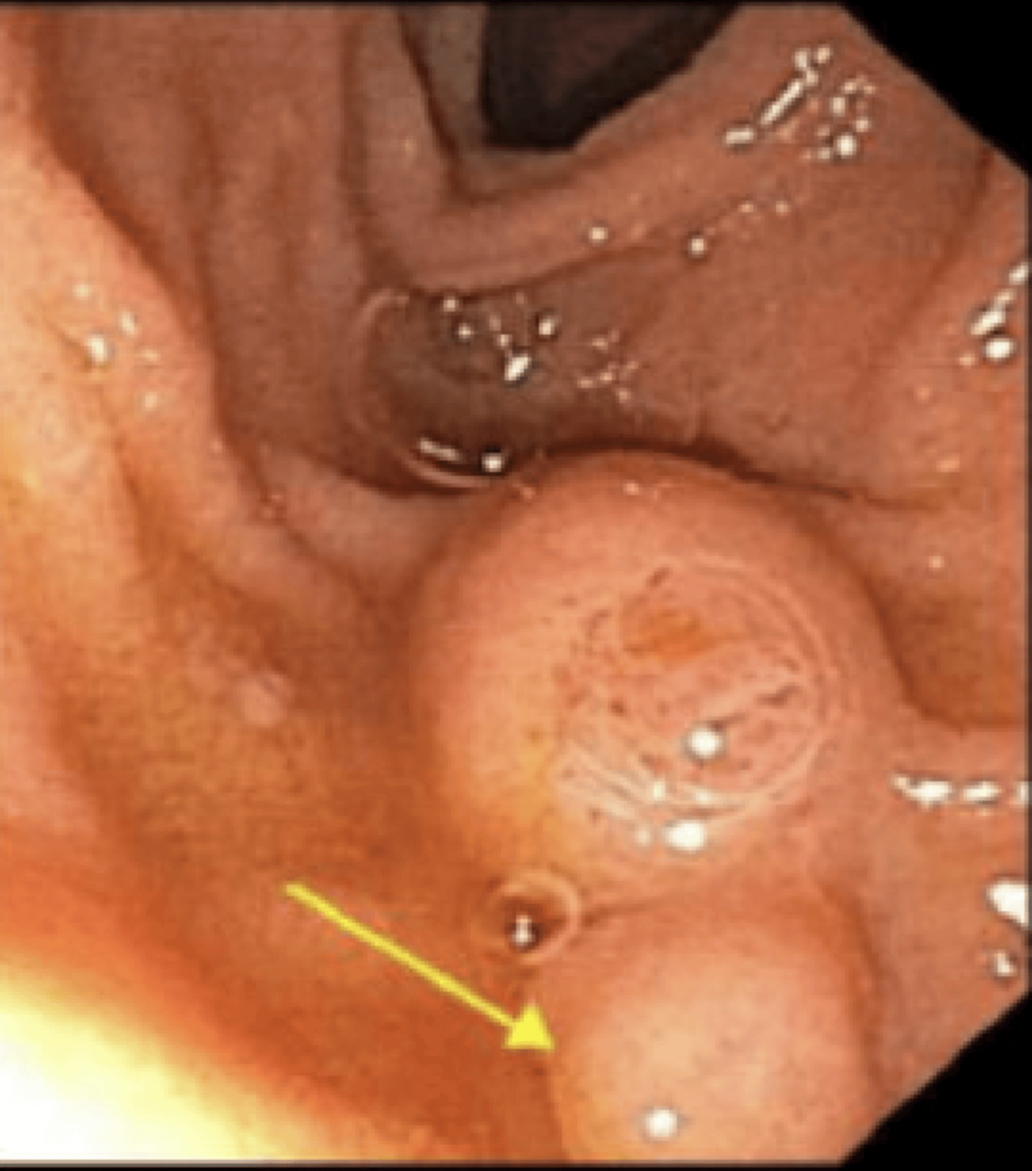
EGD showing a nodule in the second portion of the duodenum EGD: Esophagogastroduodenoscopy

**Figure 2:**
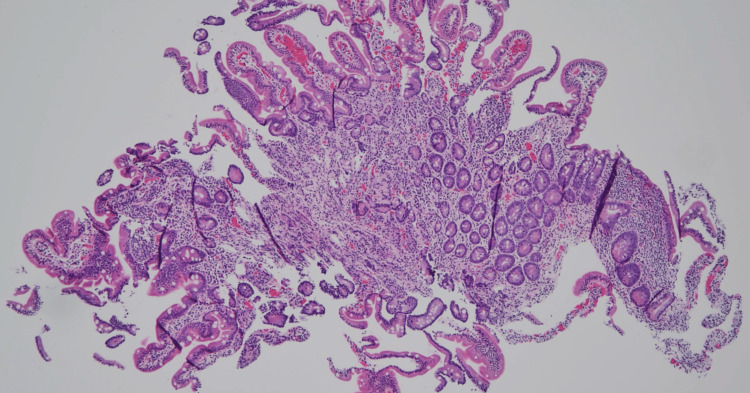
Biopsy of the nodule showing duodenal mucosa with submucosal nodules composed of benign glands and smooth muscle bundles, consistent with adenomatous hyperplasia

A 58-year-old lady, originally from Guatemala, presented to the clinic with complaints of heartburn and intermittent dysphagia for the last three years. Her past medical history is significant for synchronous endometrial and ovarian cancer treated with hysterectomy and salpingoophorectomy. The symptoms were not relieved with antacids and PPI therapy. She did not report any history of unintentional weight loss. Given that the persistent heartburn was unresponsive to PPI, esophagogastroduodenoscopy (EGD) was performed. EGD showed a 10 mm submucosal nodule in the duodenum that was biopsied (Figure 1). Endoscopic ultrasound (EUS) evaluation could not be done as it was technically difficult to pass the EUS probe to the second portion of the duodenum. Biopsy showed duodenal mucosa with submucosal nodules composed of benign glands and smooth muscle bundles, consistent with adenomatous hyperplasia (Figure 2).

**Figure 3:**
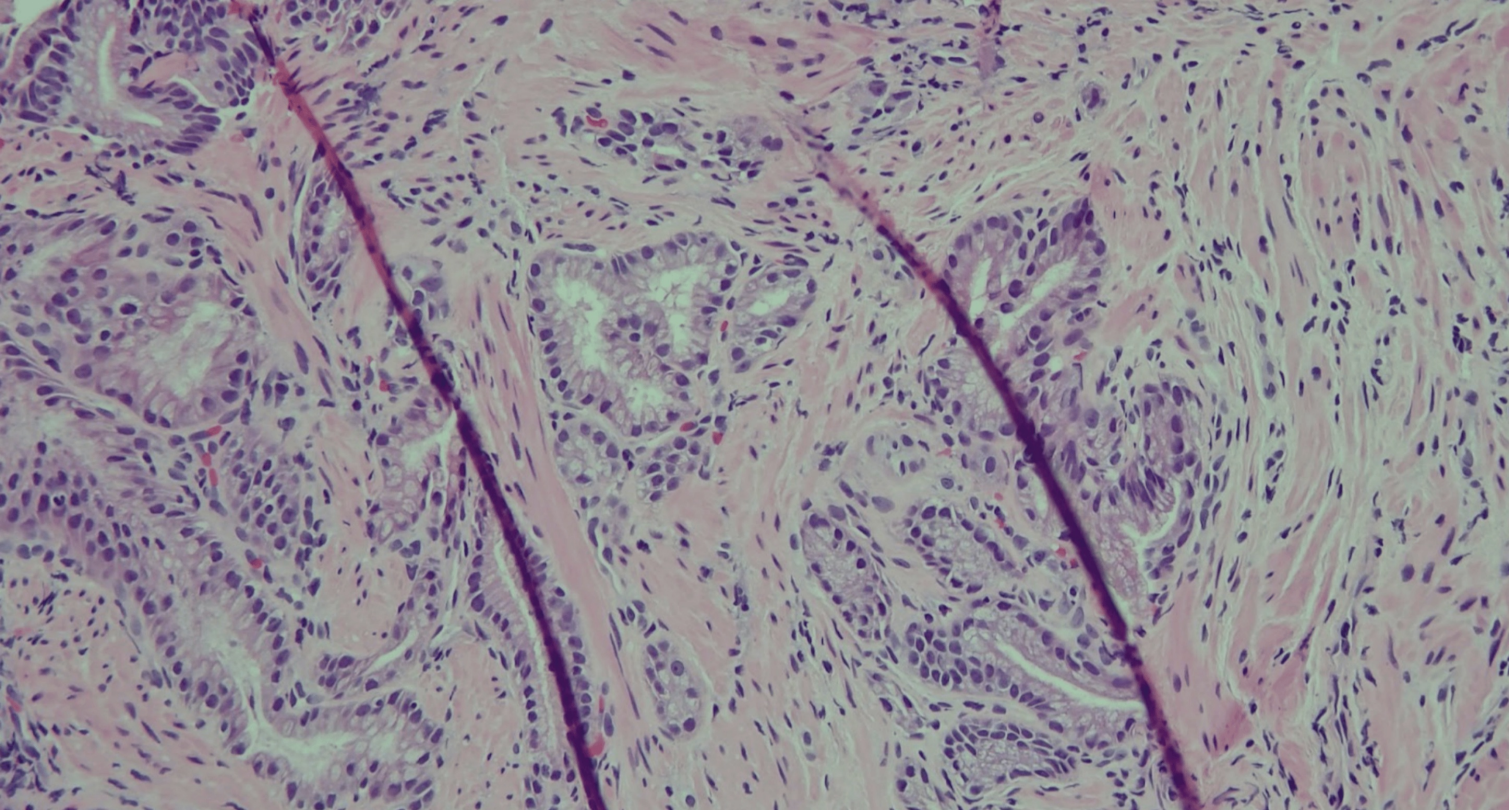
Histology of the nodule showing nodular disorganization with the proliferation of ducts, glands, and smooth muscle cells

**Figure 4:**
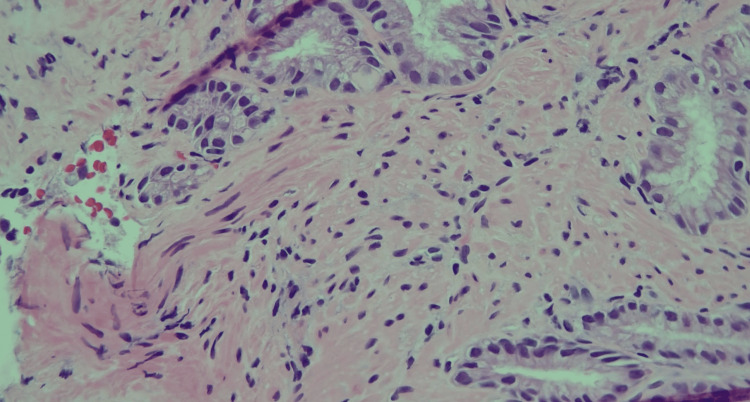
Magnified histologic image showing nodular disorganization

Histological examination showed nodular disorganization with the proliferation of ducts, glands, and smooth muscle cells (Figures 3, 4). Awaiting the pathology results, the patient was started on famotidine 20 mg daily. Since the PPI did not provide symptomatic improvement, it was deferred. The patient reported improvement in symptoms with famotidine. Therefore, famotidine was continued along with general anti-reflux measures, including weight loss, loose-fitting clothes, abstaining from trigger foods, raising the head of the bed when sleeping, and waiting three hours after dinner before going to bed. The patient’s symptoms improved, and the repeat EGD one year later showed no increase in the size of the mass.

## Discussion

The submucosal layer of the first part of the duodenum has abundant Brunner’s glands, which are acinotubular exocrine glands. These glands progressively decrease in size and number in the distal duodenum and are majorly populated in the duodenal bulb and proximal duodenum. The primary function of Brunner's gland is mucin production, which acts as an alkaline protective barrier from the acidic content of the stomach [4]. After Cruveilhier first described the case of benign duodenal Brunner’s gland adenoma in 1835 [6], over 100 sporadic reports have been recorded. BGH, also called adenoma or hamartoma, is a relatively rare lesion in the duodenum [5]. In an analysis of over 25,000 upper gastrointestinal tract biopsies, duodenal polyps were merely present in 1.6% of them, and 6.9% were cases of BGH [7]. Levine et al. reported the largest case series on the clinical characteristics of Brunner's gland hamartomas with a median age of 56 years at diagnosis of BGH and a higher incidence in female patients (female: male, 5:4) [8]. Our patient, who is a 58-year-old woman, is quite close to the mean age reported in the case series.

The exact mechanism behind BGH remains unclear. The primary hypothesis suggests that BGH can be due to an adaptive response triggered by increased gastric acidity, chronic Helicobacter pylori infection, or pancreatic exocrine insufficiency [2,5,9]. Another school of thought suggests that loss of alkaline protection usually provided by the exocrine pancreas could lead to compensatory hyperplasia of the Brunner glands, which causes an increased production of mucus and alkali [5]. Most individuals with BGH do not experience noticeable symptoms. However, some may develop non-specific complaints such as indigestion, bloating, flatulence, or signs of obstruction and unintended weight loss. In rare cases, gastrointestinal bleeding may also occur [10]. Rarely, patients can present with gastric outlet obstruction [11]. Only two patients so far have been reported in medical literature who presented with duodenal intussusception, perhaps due to duodenal fixation to the posterior abdominal wall [9]. Some patients report diarrhea, which is thought to be caused by duodenal motor disturbances [12]. Our patient presented with chronic heartburn with intermittent dysphagia unrelieved by proton pump inhibitors.

The differential diagnosis for a duodenal submucosal nodule includes gastrointestinal stromal tumors, leiomyomas, lipomas, pancreatic rests, and neuroendocrine tumors. On imaging, particularly EUS, these lesions may appear similar. Histopathological evaluation is critical in distinguishing BGH from these potentially malignant lesions, especially given the overlap in presentation and location. In our case, the absence of cytological atypia, necrosis, or mitotic activity on biopsy confirmed the diagnosis of BGH, effectively ruling out malignant or pre-malignant conditions.

BGH is usually a chance discovery and presents an incidental finding during endoscopic examinations. It usually presents as a duodenal submucosal nodule. However, when it is large, non-invasive imaging like computed tomography (CT) with an IV contrast imaging can show a duodenal growth separate from pancreatic parenchyma [10]. However, distinguishing BGH from malignant lesions can be challenging on imaging or histopathology. EUS is the gold standard imaging modality for diagnosing submucosal BGH. Matsushita et al. describe BGH on EUS as a hypoechoic, heterogeneous mass with indistinct margins and cystic components, typically confined to the duodenal submucosa and muscularis propria [13]. EUS has proved effective in defining the origin, extent, and vascularity of submucosal duodenal lesions [14]. The effective role of EUS for BGH has been backed by multiple recent cases due to consistent findings of hypogenic mass in the duodenum [15]. However, in the majority of cases, diagnosis is done by histopathological evaluation of a duodenal mass, which is seen on EGD. Typically, BGH appears as mucosal protrusions or polyps on EGD [10]. The differentials at that time can be large, including adenomatous polyps, leiomyoma/leiomyosarcoma, gastrointestinal stromal tumors, or pancreatic tumors. The biopsy showing the proliferation of Brunner’s glands with interspaced smooth muscle bundles and the absence of cytological atypia, necrosis, or invasion helps in ruling out malignant etiology and confirming the diagnosis of BGH [16]. In our case, there was a 10 mm submucosal nodule in the duodenum, and histopathological evaluation helped confirm the diagnosis of BGH.

As of now, there is no consensus on the management of BGH. Medical treatment mainly involves the management of gastric hyperacidity, which is a known cause of BGH, but the regression of BGH is rare [3]. However, periodic EGD surveillance is required to ensure it is not increasing in size. To prevent potential complications like bleeding, significant anemia, obstruction, or intussusception, endoscopic or surgical excision is often recommended [17,18]. Endoscopic polypectomy is generally the preferred method due to its minimally invasive nature and cost-effectiveness compared to open surgery [17]. However, the feasibility of this approach depends on factors such as lesion size, location, and whether it is pedunculated [18]. Open surgical resection is typically considered when endoscopic snaring is unsuccessful or for larger lesions [2]. Postoperative outcomes are generally favorable, with no reported cases of recurrence [2]. Given the rarity of BGH, especially in symptomatic presentations, and the absence of consensus on optimal treatment, this report contributes valuable insight into its clinical spectrum and management. A summary of similar reported cases is provided to contextualize this case and support clinical decision-making (Table 1). In our patient, we did not proceed with surgical management because the patient responded well to medical management (famotidine) and patient preference. Repeat EGD surveillance showed no increase in the size of the BGH.

**Table 1:** Summary of reported cases of Brunner’s gland hyperplasia: clinical presentation, management, and outcomes

Author / Year	Age / Sex	Symptoms	Lesion Size	Management	Outcome/Follow-Up
Levine et al., 1992 [8]	56 (median)	GI bleeding, pain, obstruction	Variable	Endoscopic / Surgical	Favorable, no recurrence reported
Matsushita et al., 2011 [13]	65/F	Obstruction	20 mm	Endoscopic resection	Asymptomatic at six-month follow-up
Liang et al., 2020 [18]	81/F	GI bleeding, bloating, dyspepsia	10 mm	Endoscopic removal	No recurrence at six-month follow-up
Current Case	58/F	Heartburn, dysphagia	10 mm	Medical (famotidine), EGD f/u	Symptom resolution, stable at 1 year

## Conclusions

BGH is a rare yet clinically significant cause of refractory gastrointestinal symptoms such as dyspepsia and abdominal pain. This case highlights the importance of maintaining a broad differential diagnosis and pursuing thorough diagnostic evaluation, including histopathological confirmation, to rule out malignancy. While BGH is typically benign and often discovered incidentally, its ability to mimic more serious pathology underscores the need for clinical awareness. Management can range from medical acid suppression to endoscopic or surgical resection, depending on symptom severity and lesion characteristics. Our case reinforces the importance of individualized treatment strategies and the recognition that not all lesions require intervention. Prompt and accurate diagnosis is essential to avoid unnecessary procedures and reduce the risk of overtreating a benign condition.
